# Evaluation of the Sensitivity of Various Reinforcement Patterns for Structural Carbon Fibers to Open Holes during Tensile Tests

**DOI:** 10.3390/polym13244287

**Published:** 2021-12-07

**Authors:** Elena Strungar, Dmitrii Lobanov, Valery Wildemann

**Affiliations:** Center of Experimental Mechanics, Perm National Research Polytechnic University, 614990 Perm, Russia; cem.lobanov@gmail.com (D.L.); wildemann@pstu.ru (V.W.)

**Keywords:** carbon-fiber composite, 3D reinforced composite, digital image correlation

## Abstract

This paper is devoted to the experimental study of polymeric composite specimens, with various types of reinforcement, in order to evaluate the breaking strength of specimens with open holes when undergoing uniaxial compression and tensile tests. Four types of interlaced 3D woven preforms were considered (orthogonal, orthogonal combined, with pairwise inter-layer reinforcement, and with pairwise inter-layer reinforcement and a longitudinal layer), with a layered preform used for comparison. Tensile tests of solid specimens without a hole, under ASTM D 3039, and of specimens with an open hole, under ASTM D 5766, were carried out using the Instron 5989 universal electromechanical testing system. Movements and strains on the specimen surface were recorded using a Vic-3D contactless optical video system and the digital images correlation method (DIC). For all the series of carbon fiber tension specimens, strain and stress diagrams, mechanical characteristics, and statistical processing for 10 specimens were obtained. The paper evaluated deformation fields for certain points in time; the obtained fields showed an irregular distribution of deformation and dependency on types of reinforcing fibers. A coefficient of strength variation is introduced, which is defined as a ratio of the ultimate stress limits obtained on solid samples with and without open holes. Within the framework of ASTM D 5766, when calculating the ultimate stress, the hole is not taken into account, and the paper shows that for certain structures a hole cannot be excluded. The hole size must not be neglected when calculating the ultimate stress.

## 1. Introduction

An obvious disadvantage of conventional polymeric composite materials, in the form of fabric-based laminates, is their relatively low inter-layer strength. Nowadays, spatial-reinforced fillers or 3D fabrics are proposed to prevent this. Multi-layered carbon fabrics of various weavings are used as reinforcement materials for carbon fibers, operating under complex conditions of high-speed aerodynamic flows, vibrations, and high temperatures. Three-dimensional woven composites, developed using conventional weaving technology, have better characteristics of thickness and a higher impact strength as compared with conventional layered composites [1,2,3,4].

Researchers have achieved great success in studying the mechanical properties of composite laminates with an open hole and have recently started to study 3D textile composites [5,6,7]. The work [2] studies the failure mechanisms of 3D orthogonal woven composites made of E-glass and epoxy resin with drilled and molded holes using X-ray technology. The authors of [3,4] analyzed the fatigue durability of 2.5D woven composites with a central hole at room and elevated temperatures, and these papers also studied plates with open holes of various diameters during tensile tests.

Composites are used for complex structures where various elements are bolted or riveted. Thus, understanding and predicting the mechanical behavior of composite elements of structures with holes have become necessary in order to design complex irregular structures. Stress concentration may lead to failure and significantly reduced strength. The analysis of the effects of stress concentration on the behavior of composite materials is an important topic; they are believed to cause a relatively high strength reduction as compared with composite objects without holes [8]. When structural parts are subjected to various loads during operation, high-stress concentration may appear near the hole and cause a reduction in mechanical characteristics. The studies [9,10,11,12] clearly show that the sensitivity of the composites in the presence of holes corresponds to an increase in damage within the composite until final failure. Mechanisms forming the damage zone in composites include many interacting types of damage: matrix fractures, laminations, fractures of transverse layers, etc. Averbukh and Madakur [13] gave an overview of the types of damage, which depend on placement, material properties, and geometry. Lamination is one of the primary defects that occur when drilling holes in composite objects and can be an important limiting factor in using composite materials since the drilling process causes micro-fractures that are a concentrated source of high stress.

Due to the very complex damages to composite structures with holes, all failure models contain approximated solutions or suggestions that have been more or less confirmed by experimental observations [14,15,16,17,18,19]. Therefore, experimental methods are required to improve models and confirm their results. Various experimental methods are available to detect localized damages caused by mechanical loading in composites with holes [11,20,21,22,23].

When measuring mechanical characteristics, devices are generally applied to detect movements using two points. Such measurements do not provide complete information on the distribution of the deformations. This information can be obtained when using optical methods for measuring deformation. One such method, which is widely used in connection with the development of computer-based imaging techniques, is the correlation of digital images. This method is based on analyzing the area of interest using a couple of digital photos made before and after specimen deformation [24]. Deformation measured by a strain gauge reflects strain changes in a specific point in the material, while deformation measured by DIC pays more attention to strain changes across a complete field. The strain-gauge method is more accurate in detecting some sensitive areas, while the DIC method is most suitable for the overall monitoring of deformation along the entire surface of the composite specimen. When the material fails, the strain gauge can be most accurate in finding the failure moment, while it is harder to find it using DIC [4]. Taking into account that the behavior of composite structures may differ in various types of tests, the work [25] carried out an experimental study of shear properties in spatial-reinforced composites using the Iosipescu ASTM D5379. Recommendations were made to obtain and mathematically model experimental data using a contactless optical video system.

The paper [25] shows the features of using the Iosipescu ASTM D5379 for a composite with layer-to-layer reinforcement.

This work is a continuation of a comprehensive study of 3D woven carbon fiber-reinforced plastics under different types of loading. Previously, the authors in works investigated a total of eight structures (orthogonal, orthogonal conjoined, with layer-to-layer reinforcement, with layer-to-layer reinforcement and a longitudinal layer, with layer-to-layer conjoint reinforcement, and with through layer-to-layer reinforcement) [6,7,8]. In the considered work, five configurations were selected, one type from each pair. Such reinforcement schemes were studied as model ones for studying the implementation of mechanical properties and subsequent transfer (transition) to the modeling, design, and manufacture of high-load and medium-load structural elements for critical purposes. Through the analysis of the mechanical strength of fabric-based composites, the performance advantages of 3D reinforced composites are further derived [26,27].

The main aim of this article is an evaluation of the sensitivity of various reinforcement patterns for structural carbon fibers to open holes during tensile tests. This study is required to understand the role of composite damage mechanisms, compare the performance of various reinforcement types, and evaluate the sensitivity to open holes. Damages occurring around the holes, and the nature of their distribution along the entire surface, were studied using the Vic-3D contactless optical video system and the DIC method of correlating digital images.

## 2. Materials, Experiments and Methods

### 2.1. Material

**Figure 1:**
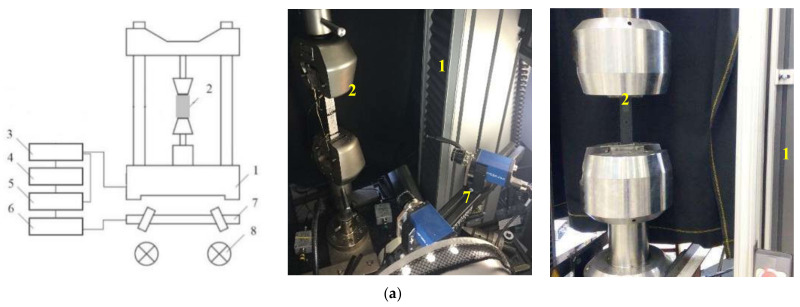

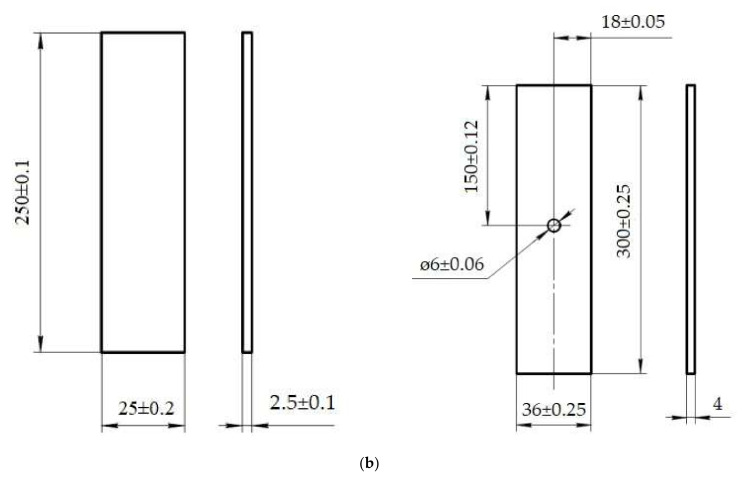
Principled testing scheme for the uniaxial tension of specimens (**a**): 1—test machines, 2—sample installed in the grips, 3—test system controller, 4—PC from which the machine is controlled, 5—synchronization block, 6—PC from which the video system is controlled, 7—cameras installed on tripod, 8—backlight system. Scheme of specimens without an open hole and with an open hole (**b**).

A spatially reinforced carbon fiber composite, based on T26 epoxy resin and AKSA A-49 carbon fiber, was used as the material for this study. A number of mechanical tests were carried out for the uniaxial compression of composite specimens with a round concentrator in the form of a hole along the symmetry axis in the specimen (as per the specimen in Figure 1b, W1 = W2 = 15 mm and L1 = L2 = 147 mm). Preforms of stripe specimens were made using 3D weaving by various weaving methods (Types 1–4), as well as layered preforms (Type 5). Four weaving patterns of 3D woven preforms were considered: orthogonal (Type 1); orthogonal combined (Type 2); with pairwise inter-layer reinforcement (Type 3); and with pairwise inter-layer reinforcement and a longitudinal layer (Type 4). In the central part of the rectangular specimens, which were 300 × 36 × 4 mm in size (L × W × h) (under ASTM D 5766), there was a concentrator shaped as a single open hole, 6 mm in diameter, and specimens without a concentrator were 250 × 25 × 4 mm (L × W × h) in size (ASTM D 3039). Since holes were made in the specimens by drilling, defects could form around the holes. Using the Carl Zeiss SteREO Discovery V12 stereo microscope, it was found that damages represented by small shears of the binder were located on outer surfaces around the edge of the holes. The overall extent of plate damage was on average less than 1% of the working volume.

Testing schemes (a) and sketches of specimens (b) without and with an open hole are given in Figure 1. Ten specimens in each group were tested.

### 2.2. Equipment

Experimental studies were carried out using the large-scale research facilities «Complex of testing and diagnostic equipment for studying properties of structural and functional materials under complex thermomechanical loading» at the PNRPU.

The tests were carried out at the Center of Experimental Mechanics of the Perm National Research Polytechnic University in the city of Perm, Russia.

Tensile tests were carried out using the Instron 5989 (± 600 kN) and Instron 5982 (± 100 kN) universal electromechanical testing systems with a movable grip speed of 2 mm/min (Figure 1). The loading was recorded by a load cell up to 100 kN and 600 kN. The loading measurement accuracy is 0.5% of the measured value within 0.2–1% of the nominal capacity of the load cell.

Movements and strains on the specimen surface were recorded using a Vic-3D contactless optical video system and the digital images correlation (DIC) method. Video recording of the deformation process was done using Limess 2,0/28/0901 lenses. The filming speed was 15 frames per second with an installed camera resolution of 4.0 MPa. Using a contactless optical video system requires the synchronization of experimental data with the loading process.

The testing system controller was connected to a 16-bit high-speed NI USB-6251 ADC unit supplied with a signal for traverse loading and movement (according to the integrated sensor). The Vic-3D video system was connected to the ADC unit.

### 2.3. Study Methods

Tensile tests of solid specimens without a hole were carried out under ASTM D 3039 using Instron 5982 (± 100 kN), and tensile tests of specimens with an open hole were carried out under ASTM D 5766 using Instron 5989 (± 600 kN).

The tensile testing results of specimens with an open hole, and without a hole, were used to find the respective mechanical characteristics. The comparison was based on the maximum stresses.

Before testing and before loading, a fine coating must be applied to the surface. The latter ensures an accurate determination of movements and increases the contrast of the surface. A number of black and white points were applied to the surface of the specimen using spray paint. In the DIC method, a zero-mean normalized sum of squared difference criterion was selected since it is the least sensitive to illumination changes during tests. During post-processing by the Vic-3D system, the strain components were calculated using the finite strain tensor in the Lagrange representation $\varepsilon_{ij}=\frac{1}{2}\;\left(u_{i,j}+u_{j,i}+u_{k,i}u_{k,j}\right).$ The Oy axis is directed along the specimen (along the elongation axis), and the Ox axis is perpendicular to the loading axis in the specimen plane. When building loading diagrams, an additional software module of the virtual extensometer video system was used to track a mutual shift between two points on the surface of the specimen in relation to the applied force.

The contactless optical system’s accuracy is determined by the technical characteristics of lenses and digital cameras, namely the matrix sensitivity, resolution, and permissible frame frequency. The accuracy of obtained experimental data is also influenced by the surface of the specimen and the configuration and calibration of cameras [24]. Upon test results given in [6], a conclusion was drawn that by using the Vic-3D digital optical system, it is possible to determine the strain on a fixed base with an accuracy comparable to the data on a suspended longitudinal strain sensor whose maximum possible deviation from the measured value is 0.15%.

### 2.4. The Method of Using Contactless 3D Digital Optical Systems

When processing digital photos, vector shifts are not calculated in each individual point of the image (in pixels), but rather sampling is done in the area of study in small local sub-areas or, in other words, subsets (correlation areas) X × X in size [24]. The subset size has a significant effect on the accuracy of the correlation analysis and the detail of movement fields and strains along the surface of the studied object, as well as the size of the zone occurring at the edge or near stress concentrators (holes, inclusions, fractures, defects). The step defines the distance in pixels between points (central pixels of the subset) that are analyzed during mathematical processing [24].

The sub-area size (Χ) and increment (ΔΧ) were selected as per the filing conditions, calibration results of the stereo system, as well as the dependency on geometric parameters of the study object and structural specifics of the specimen materials.

**Figure 2:**
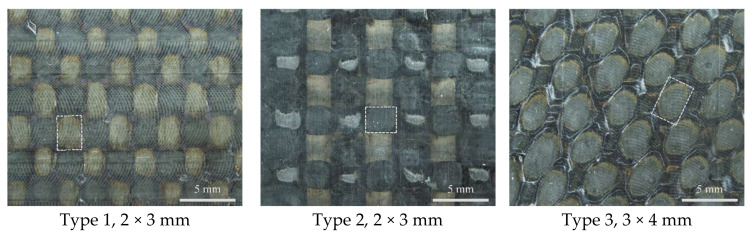

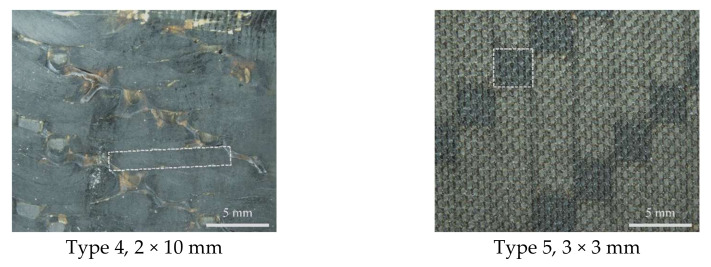
Photo of the surface of samples of three-dimensional-reinforced composites of various weaving patterns with a highlighted structural element (δ).

This paper takes into account structural features occurring on the surface of a composite material, which were used to select increment ΔΧ. The value of increment ΔΧ depends on the size of the structural element of the material (δ) that was found from a photo made using the Carl Zeiss SteREO Discovery V12 stereomicroscope (Figure 2).

Since the numerical image processing parameters have a significant influence on the results of movement fields and strains, the presentation of results obtained by the digital image correlation method must include the subset size (X), increment (ΔX), number of points (N) for the area under study, size of the composite material structural element (δ), and pixel size (s) [28,29,30] (Table 1).

It should be noted that the selection of the parameters of the correlation analysis was done taking into account the material’s structural irregularity, which may affect the recording scale of the strains. To change structural strains, a smaller increment must be set that takes into account the structure of the material [28,29,30].

The studies show that parameters should be selected taking into account the size of the material’s structural irregularity. Since the parameters of numerical image processing have a substantial effect on the results, the conclusion was drawn that when presenting these data, the following parameters must be indicated: subset size, increment, number of points for the area under study, size of the composite material structural element, and pixel size.

## 3. Test Results and Discussion

**Figure 3:**
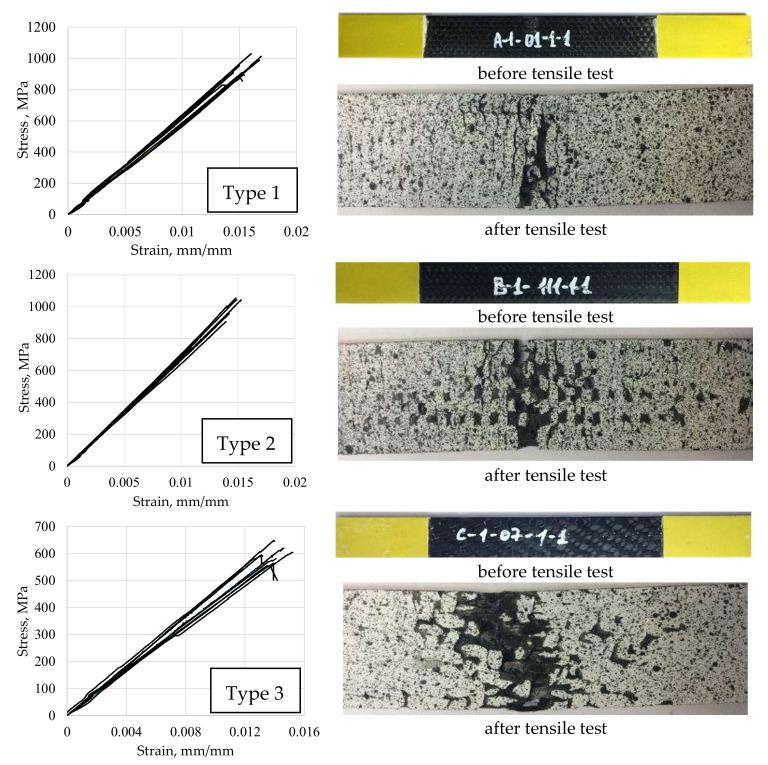

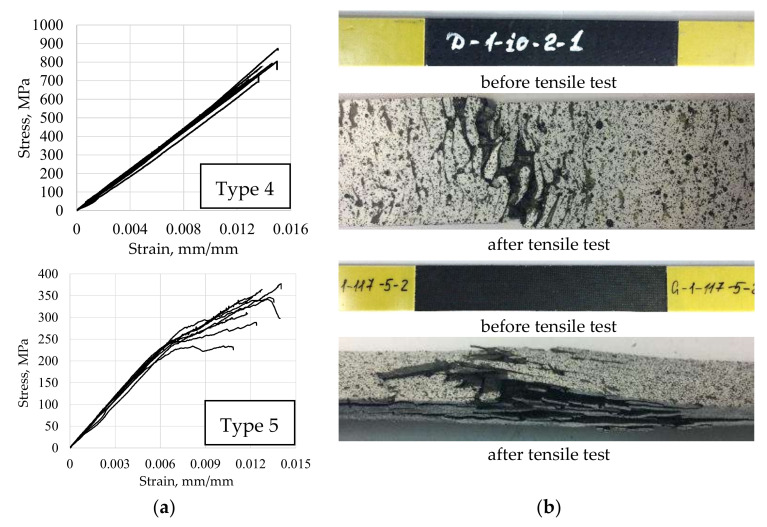
Deformation diagrams (**a**) and view of CFRP specimens of various reinforcement schemes before and after (**b**) the uniaxial tensile test (according to ASTM D 3039).

For all the series of carbon fiber tension specimens tested under ASTM D 3039, strain and stress diagrams (Figure 3), the ultimate strength, and statistical processing for 10 specimens (Table 2) were obtained.

According to the test results, it can be noted that in accordance with the classification of failure types according to ASTM D 3039, all tested samples were analyzed using a three-part failure mode code (the first character means Failure Type; the second character means Failure Area; and the third character means Failure Location).

Type 1 and Type 2 CFRP samples were destroyed by the LAT (L, Lateral; A, At grip/tab; T, Top) and LGM (L, Lateral; G, Gauge; M, Middle) mechanisms. The Type 3 samples were destroyed by the LGM mechanism. Samples of Type 4 were destroyed by the LGM and AGM (A, Angled; G, Gauge; M, Middle) mechanisms. Samples of Type 5 were destroyed by the DGM (D, edge Delamination; G, Gauge; M, Middle) mechanism.

Analyzing the deformation diagrams for samples of Type 5, it can be observed that when 65–70% of the ultimate strength was reached, the slope of the curves changed.

The authors attribute this phenomenon to the appearance of local damage in the specimen in the form of inter-layer delamination, which subsequently developed to the macrolevel and led to the complete destruction of the specimen.

**Figure 4:**
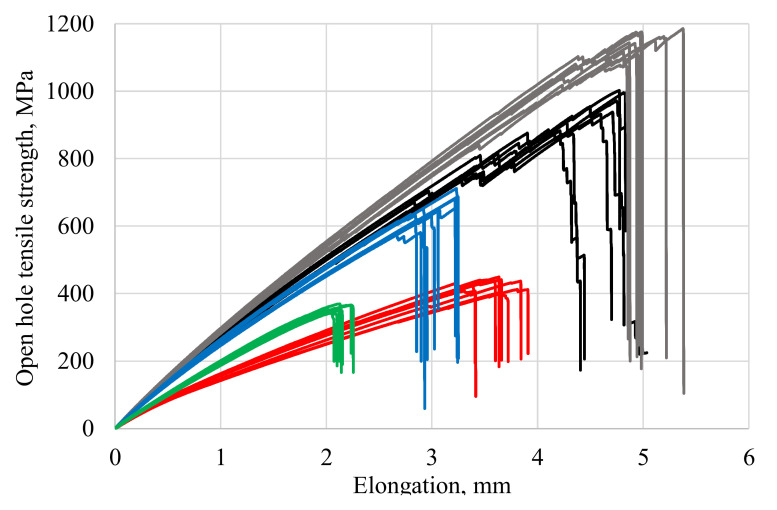
Loading diagrams of CFRP specimens with an open hole in tension (according to ASTM D 5766) with different reinforcement schemes: Type 1—black curves; Type 2—grey curves; Type 3—red curves; Type 4—blue curves; Type 5—green curves.

**Figure 5:**
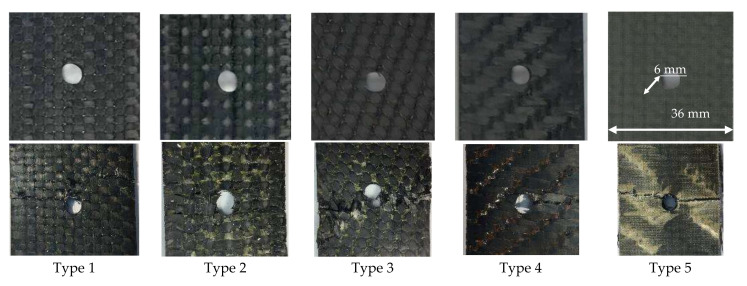
Photographs of CFRP samples in the vicinity of the concentrator before and after tensile tests.

Figure 4 presents loading diagrams for specimens with an open hole tested under ASTM D 5766, which are characteristic of each reinforcement type. The tensile and specimen tests, with an open hole, show that polymeric composite specimens with orthogonal (Type 1) and orthogonal-combined (Type 2) interweaving schemes have a high ultimate load as compared with specimens with inter-layer reinforcement (Type 3, Type 4) and layered specimens (Type 5). For all carbon fiber specimens with different reinforcement types, the loading diagrams show breaks related to structural failure. Figure 5 gives photos of failed specimens of all reinforcement types with characteristic damages in the area of the hole. In general, almost all curves on the loading diagram (Figure 4) are linear. In some cases, at a later loading stage, the curves show obvious breaks caused by permanent local damages and the reduced bearing capacity of the structure (Types 1, 2, and 3). By analyzing the failure damage of carbon fiber specimens with a hole, it can be noted that Type 1 specimens under the ASTM D 5766 (analog ASTM D 3039) classification failed predominantly under the LGM mechanism. Type 2 specimens failed under various mechanisms: half of them failed under the LGM mechanism, while others failed under the OGM (O, Other; G, Gauge; M, Middle), OMV (O, Other; V, Various; M, Middle), SGM (S, long. Splitting; G, Gauge; M, Middle), and OUU (O, Other; U, Unknown; U, Unknown) mechanisms. For Type 3 specimens, the LGM and AGM mechanisms prevailed, while the MGM (M, Multi-mode; G, Gauge; M, Middle) and AGM mechanisms prevailed for Type 4 specimens. The Type 5 specimens failed under a single mechanism (MGM) (Figure 5). The destruction mechanism (Type 5) can be classified as multiple inter-layer delamination in the area of an open hole (concentrator) with a subsequent detachment of the surface layers.

Analyzing the deformation diagrams, it can be observed that specimens of Type 1 and Type 2 are characterized by disruptions in the loading diagram in the ascending section. For Type 1 samples, breakdowns in the ascending section of the diagram were recorded when reaching from 60 to 70% of the *F^OHTu^* values; for Type 2 samples, breakdowns were recorded when the *F^OHTu^* reached 70 to 80%.

The authors attribute these phenomena to the fact that the reinforcement system rotates in the direction of load application, which leads to local fractures in the matrix–fiber system, followed by redistribution of the load to neighboring nodes, and so on until the next local failure or complete destruction of the sample. It is also worth noting that the Type1 and Type 2 specimens have the largest deformation margin.

Test results for each group of specimens gave the mechanical characteristics presented in Table 2, mean values (x), mean-square deviations (S), and variation coefficients (V).

It should be noted that ultimate open hole tensile strength *F^OHTu^* refers to the ratio between the maximum loading and the initial cross-sectional area of the tested specimen (excluding the hole). The maximum load *P_max_* refers to the maximum load prior to failure (Formula (1)).

(1) $$F^{OHTu}=\frac{P_{max}}{hw\prime }$$

**Figure 6:**
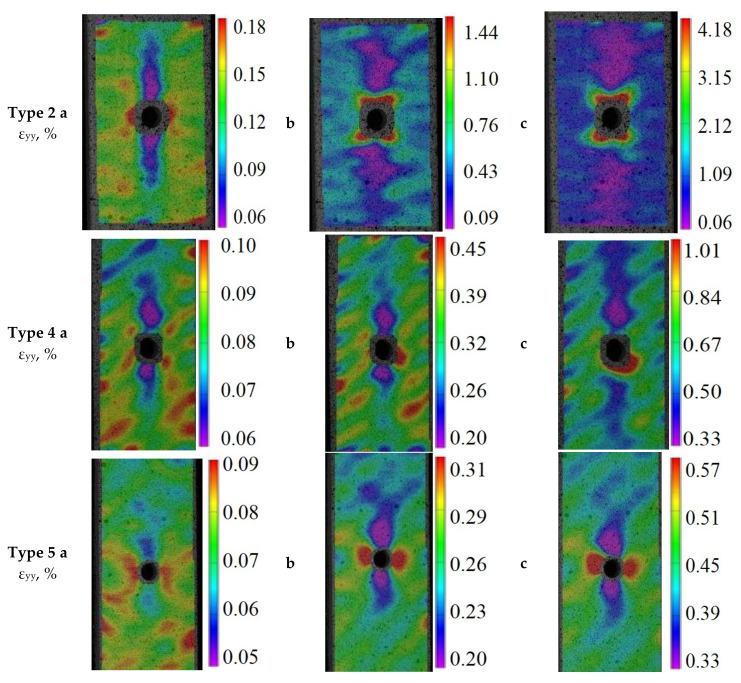
Fields of longitudinal strains ε_yy_ of the surface of specimens of reinforcement types 2, 4, and 5 for a loading of 10% (**a**), 40% (**b**), and 70% (**c**) of the maximum value *F^OHTu^*.

To evaluate irregular strain fields obtained using the video system, specimens with reinforcement types 2, 4, and 5 were considered, which have the highest (Type 2), medium (Type 4), and minimal (Type 5) ultimate loading (Figure 6). The Oy axis is directed along the specimen (along the elongation axis), while the Ox axis is perpendicular to the loading axis in the specimen plane.

The obtained data show that the concentration of stress on the edge of the specimen hole was gradually becoming prominent as the loading developed. The areas of maximum and minimal strain in the specimens appeared on the left/right side and upper/lower side of the holes, respectively, which resulted in a symmetric distribution of the strain field around the edge of the hole. However, at the early stage of loading, strains were not symmetrical. Strains in the upper right part of the specimens were much larger than in other parts, but this phenomenon disappeared at a later loading stage.

**Figure 7:**
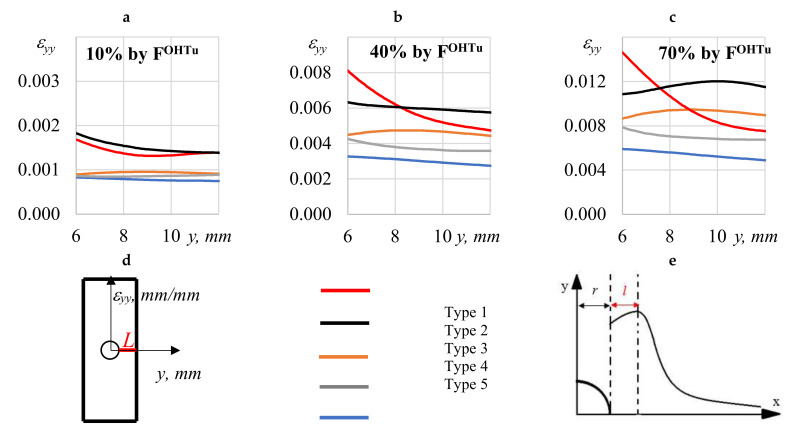
Profiles (ε_yy_) on the sample surface at specific stress levels of 10% (**a**), 40% (**b**), and 70% (**c**) of the ultimate stress *F^OHTu^*. Profiles (ε_yy_) on the sample surface (**d**). The schematic distribution of the strain (ε_yy_) (**e**).

For a more detailed analysis, the distribution of longitudinal stress ε_yy_ was evaluated on the specimen surface (Figure 7d) along line L drawn from the hole to the plate edge (12 mm away). Strain diagrams were built at specific stress levels of 10% (Figure 7a), 40% (Figure 7b), and 70% (Figure 7c) of the ultimate stress *F^OHTu^*.

The results show that the behavior of specimens with an open hole, with reinforcement types 1, 4, and 5, remained linear-elastic until failure. For reinforcement types 2 and 3 for higher tensile loads, the maximum strain shifted to the plate edge some distance from the hole. The schematic distribution of the strain *ε_yy_* from the hole to the plate edge along line L is shown in Figure 7e. Similar experimental results were obtained by researchers in [16,31,32].

Table 3 shows the correspondence between the maximum longitudinal strains (*ε_yy_, max*) and the distance from the hole (l), depending on the material structure. The higher the applied load, the higher the maximum strain. The maximum longitudinal strain *ε_yy_, max* = 1.20% was measured for the structure of specimen Type 2.

It was observed that the higher the applied loading, the higher the maximum strain and the wider the distribution. At a distance from the hole, the distribution of longitudinal strains (*ε_yy_, _max_*) became similar to linear-elastic. It can be suggested that there is a link between the structural element size (δ) and the pixel size (s ≤ 0.080) (see Table 1) for various reinforcement types and hole sizes (d). Since the hole of the same size (d = 6 mm) includes a number of structural elements that depend on the reinforcement pattern, the strain processes may behave differently, especially when the hole size (d) is comparable to the size of the structural element (δ).

In objects with a hole, there is an obvious decrease in the strength (bearing capacity) of the composite in comparison with the same objects without a hole. To evaluate the sensitivity of a reinforcement scheme to a hole, the authors introduced a strength variant coefficient K (Formula (2)), which is defined as a ratio between the ultimate stresses of specimens with the concentrator (*F′^OHTu^*) and the ultimate strength of a solid specimen (*σ_b_*).

It should be noted that the ultimate loading *F′^OHTu^* refers to the ratio between the maximum loading and the initial cross-sectional area of the tested specimen (given the hole, and a specimen width of 30 mm) (Formula (3)).

(2) $$K=\frac{F^{\prime OHTu}}{\sigma_{b}}$$

(3) $$F^{\prime OHTu}=\frac{P_{max}}{hw}$$

where *P_max_* is the maximum load; *h* is the specimen thickness; and *w* is the specimen width given the hole (30 mm).

Within the framework of ASTM D 5766, the calculation of the ultimate stress of a specimen with a concentrator (*F^OHTu^*) was carried out without taking into account the hole, as per Formula 2, on the assumption that a hole 6 mm in diameter, with the total specimen width of 36 mm, does not affect the bearing capacity. In this case, the strength variance coefficient $\left(K\prime \right)$ will be calculated as per Formula (4).

(4) $$K\prime =\frac{F^{OHTu}}{\sigma_{b}}$$

where *w*′ is the specimen width without taking into account a hole (36 mm).

**Figure 8:**
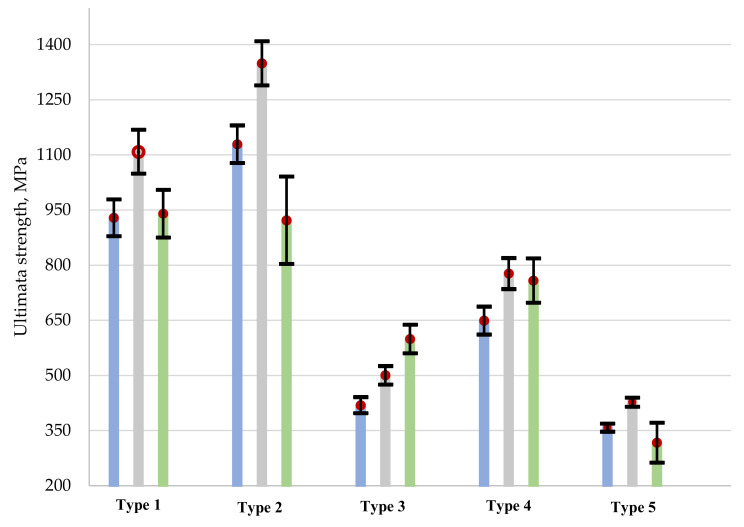
The relationship between the ultimate strength and the reinforcement type in composite specimens. The open hole tensile ultimate stress under ASTM D 5766 *F^OHTu^*—blue line; the open hole tensile ultimate strength of specimens with the concentrator *F*′*^OHTu^*—grey line; ultimate stress of solid specimen *σ_b_*—green line.

In this manner, to compare the obtained results, Table 4 gives mean values with the scattered tensile strength (*σ_b_*) and ultimate stresses in tensile tests of specimens with a hole, taking it into account (*F′^OHTu^*), and without a hole, taking it into account (*F^OHTu^*), as well as the strength variant coefficients *K* and *K*′ (under ASTM) for each type of specimen and reinforcement type. Figure 8 shows the relationship between the ultimate strength and the type of reinforcement in the composite specimens.

A comparison of stress concentration coefficients showed that Type 3 reinforcement has the most sensitive effect on the hole. For reinforcement types 1, 2, 4, and 5, the hole did not lead to a significant change in the ultimate stresses, which is related to the material structure and stress re-distribution occurring in the specimen. It can be also observed that the calculation of the ultimate stress *F^OHTu^* under ASTM differs from the ultimate stresses *F’^OHTu^*.

## 4. Conclusions

This work included a series of experimental studies of strength in structural carbon fibers with various reinforcement types in tension tests with and without an open hole. The obtained results were analyzed, and the effects of concentrators on the mechanical behavior of carbon fiber specimens were evaluated based on various spatial reinforcement frames. For all series of carbon fiber specimens tested for tension, stress and strain diagrams were obtained. Photos of failed specimens are presented for all reinforcement types with characteristic damages in the area of the open hole. The test results for each group of specimens gave mechanical characteristics, mean values (x), mean-square deviations (S), and variation coefficients (V).

Using a digital optical video system and the digital image correlation method, strain fields for solid specimens and specimens with an open hole were obtained during the entire loading process. For open-hole specimens, strain distribution diagrams were built in the area of the smallest cross-section and the longitudinal strain distribution on the specimen surface was evaluated. Reinforcement types having the lowest sensitivity in the presence of concentrators were identified.

Within the framework of ASTM D 5766, when calculating the ultimate stress, the hole is not taken into account; nevertheless, the paper shows that, for certain structures, a hole cannot be excluded. Therefore, it can be concluded that the hole size must not be neglected when calculating the ultimate stress.

## Figures and Tables

**Table 1:** Correlation analysis parameters for polymeric composite specimens with various reinforcement types.

Reinforcement Types	Χ, Pixel	ΔΧ, Pixel	N	δ, mm	s, mm
Type 1	43	3	20,383	2 × 3	0.083
Type 2	47	3	21,985	2 × 3	0.076
Type 3	55	5	13,650	3 × 4	0.076
Type 4	51	5	19,529	2 × 10	0.084
Type 5	55	3	18,500	3 × 3	0.081

**Table 2:** Mean ultimate strength (ASTM D 3039) and ultimate stresses (ASTM D 5766) for carbon fiber specimens of the studied reinforcement types.

Reinforcement Types	*σ_b_*, MPa	CV, %	*F^OHTu^*, MPa	CV, %
Type 1	940 ± 65	6.91	929 ± 50	5.38
Type 2	922 ± 119	12.91	1129 ± 51	4.52
Type 3	599 ± 39	6.51	419 ± 22	5.25
Type 4	758 ± 60	7.92	649 ± 38	5.85
Type 5	317 ± 54	17.03	358 ± 11	3.07

**Table 3:** The correspondence of longitudinal strains to the distance from the hole (l), depending on the material structure.

Reinforcement Types	*ε_yy, max_, %*	l, mm
Type 2	1.20	4.13
Type 3	0.95	2.97

**Table 4:** Test results on the strength variance coefficient for carbon fiber specimens with holes and without holes.

Reinforcement Types	*σ_b_*, MPa	*F^OHTu^*, MPa (by ASTM)	*F′^OHTu^*, MPa	*K*	*K*′ (by ASTM)
Type 1	940 ± 65	929 ± 50	1108 ± 60	1.18	0.99
Type 2	922 ± 119	1129 ± 51	1349 ± 60	1.46	1.22
Type 3	599 ± 39	419 ± 22	500 ± 25	0.83	0.70
Type 4	758 ± 60	649 ± 38	777 ± 42	1.02	0.86
Type 5	317 ± 54	358 ± 11	427 ± 12	1.35	1.13

## Data Availability

The data presented in this study are available on request from the corresponding author.
